# Structure of full-length wild-type human phenylalanine hydroxylase by small angle X-ray scattering reveals substrate-induced conformational stability

**DOI:** 10.1038/s41598-019-49944-x

**Published:** 2019-09-20

**Authors:** Catarina S. Tomé, Raquel R. Lopes, Pedro M. F. Sousa, Mariana P. Amaro, João Leandro, Haydyn D. T. Mertens, Paula Leandro, João B. Vicente

**Affiliations:** 1 Instituto de Tecnologia Química e Biológica António Xavier, Universidade Nova de Lisboa, Oeiras, Portugal; 2 Instituto de Biologia Experimental e Tecnológica, Oeiras, Portugal; 3 Research Institute for Medicines (iMed.ULisboa) and Department of Biochemistry and Human Biology, Faculty of Pharmacy, Universidade de Lisboa, Lisbon, Portugal; 4 EMBL Hamburg c/o DESY, Hamburg, Germany; 5 Present Address: Department of Genetics and Genomic Sciences and Icahn Institute for Data Science and Genomic Technology, Icahn School of Medicine at Mount Sinai, New York, NY USA

**Keywords:** SAXS, Molecular conformation, Enzyme mechanisms

## Abstract

Human phenylalanine hydroxylase (hPAH) hydroxylates l-phenylalanine (l-Phe) to l-tyrosine, a precursor for neurotransmitter biosynthesis. Phenylketonuria (PKU), caused by mutations in *PAH* that impair PAH function, leads to neurological impairment when untreated. Understanding the hPAH structural and regulatory properties is essential to outline PKU pathophysiological mechanisms. Each hPAH monomer comprises an N-terminal regulatory, a central catalytic and a C-terminal oligomerisation domain. To maintain physiological l-Phe levels, hPAH employs complex regulatory mechanisms. Resting PAH adopts an auto-inhibited conformation where regulatory domains block access to the active site. l-Phe-mediated allosteric activation induces a repositioning of the regulatory domains. Since a structure of activated wild-type hPAH is lacking, we addressed hPAH l-Phe-mediated conformational changes and report the first solution structure of the allosterically activated state. Our solution structures obtained by small-angle X-ray scattering support a tetramer with distorted P222 symmetry, where catalytic and oligomerisation domains form a core from which regulatory domains protrude, positioning themselves close to the active site entrance in the absence of l-Phe. Binding of l-Phe induces a large movement and dimerisation of regulatory domains, exposing the active site. Activated hPAH is more resistant to proteolytic cleavage and thermal denaturation, suggesting that the association of regulatory domains stabilises hPAH.

## Introduction

The human phenylalanine hydroxylase (hPAH) catalyzes the hydroxylation of l-phenylalanine (l-Phe) into l-tyrosine (l-Tyr). The reaction is the first step in the catabolic pathway of l-Phe/l-Tyr and proceeds to feed neurotransmitter biosynthetic pathways. In non-pathological conditions, degradation of excessive l-Phe by hPAH sustains physiological plasmatic levels of l-Phe (<120 µM)^1^. Deficiency in hPAH leads to phenylketonuria (PKU), characterised by a toxic accumulation of l-Phe and depletion of precursors for neurotransmitter biosynthesis in the central nervous system that overall result in cognitive disability and neurological impairment. Despite being the most prevalent disorder of the amino acid metabolism, the pathophysiology of PKU remains to be fully elucidated and treatment options are mostly limited to a life-long l-Phe-restricted diet^1^. PKU is caused by mutations in the *PAH* gene, most being missense mutations that affect folding, catalysis and/or regulation of the enzyme^2,3^. Understanding the structural and regulatory properties of hPAH is essential to outline pathophysiological mechanisms in PKU and delineate novel therapeutic strategies. However, the difficult manipulation of recombinant hPAH has thus far hindered its structural characterisation. hPAH is a member of the aromatic amino acid hydroxylases family (AAAH), also including tyrosine hydroxylase (hTH) and tryptophan hydroxylase (hTPH). All three members assemble as functional homotetramers, each subunit consisting of an N-terminal regulatory domain, a central catalytic domain and a C-terminal oligomerisation domain^4^. They harbor a catalytic non-heme iron coordinated by histidine and carboxylate residues and three waters, and use dioxygen and tetrahydrobiopterin (BH_4_) as cosubstrates. Structural analyses of hPAH have relied on crystal structures of truncated forms of the enzyme lacking one or two domains^5–12^ and full-length bound to BH_4_^12^, and on crystallographic and SAXS structures of the full-length rat homologue^13–15^. Phenylalanine hydroxylase assembles as a dimer of dimers through the C-terminal domain, forming a central four-helix bundle and a tetrameric core of catalytic domains. l-Phe binds to a pocket in the active site where the BH_4_ and the Fe^3+^ cofactors are nested. Regulatory domains are connected to the tetrameric core through a linker (Arg_111_-Thr_117_) and positioned above the catalytic domains, with the N-termini blocking the access to the active site. To regulate l-Phe blood levels, hPAH displays a complex net of mechanisms that involve transition between oligomeric states, conformational changes, substrate activation, cofactor inhibition and activation by phosphorylation (Ser_16_). Activation by l-Phe increases hPAH activity by ~3-fold^16^. Models for the activation mechanism have diverged on whether local or global motions occur upon l-Phe binding, and whether an allosteric binding site exists apart from the active binding site. The recognition of an auto-inhibited conformation led to the premise that activation would require the N-terminus to move away and release the active site entrance^14^. Further studies suggested that, rather than a simple displacement of the N-terminal portion, a large-scale conformational change would occur^17–22^. Jaffe and colleagues^19^ were the first to hypothesise dimerisation of regulatory domains upon l-Phe activation. Solution structural analyses of rat PAH confirmed distinct conformations for the inactive and l-Phe-activated enzymes and supported dimerisation of regulatory domains as the substrate activation mechanism^13,15^. Where regulatory l-Phe binds has been a matter of debate, with authors positing either a sole l-Phe-binding site at the catalytic pocket^23^ or an additional allosteric binding site^11,14,17,21,24–29^ at the regulatory/catalytic interface^14,17,24^ or at the regulatory dimer interface^11,28^. Dimerisation of individual hPAH regulatory domains is well-established^25,26,29,30^ and a recent crystal structure shows a symmetric homodimer with two l-Phe molecules bound at the dimer interface^11^. However, a structure of the activated hPAH is still lacking to confirm the behavior of regulatory domains in the context of the full-length wild-type protein. We have addressed l-Phe-mediated conformational changes of human phenylalanine hydroxylase and report the first structure of the allosterically activated state of human PAH. Our low resolution solution structures determined by small-angle X-ray scattering disclose a conformational transition from the inactive state (that agrees with crystallographic observations) to an active state where regulatory domains associate above the four-helix bundle. These structures, herein combined with biophysical data obtained for resting and activated states, validate the model of l-Phe allosteric activation and elucidate the regulatory mechanism of hPAH.

## Results

### Production of functional recombinant full-length wild-type human PAH

**Figure 1:**
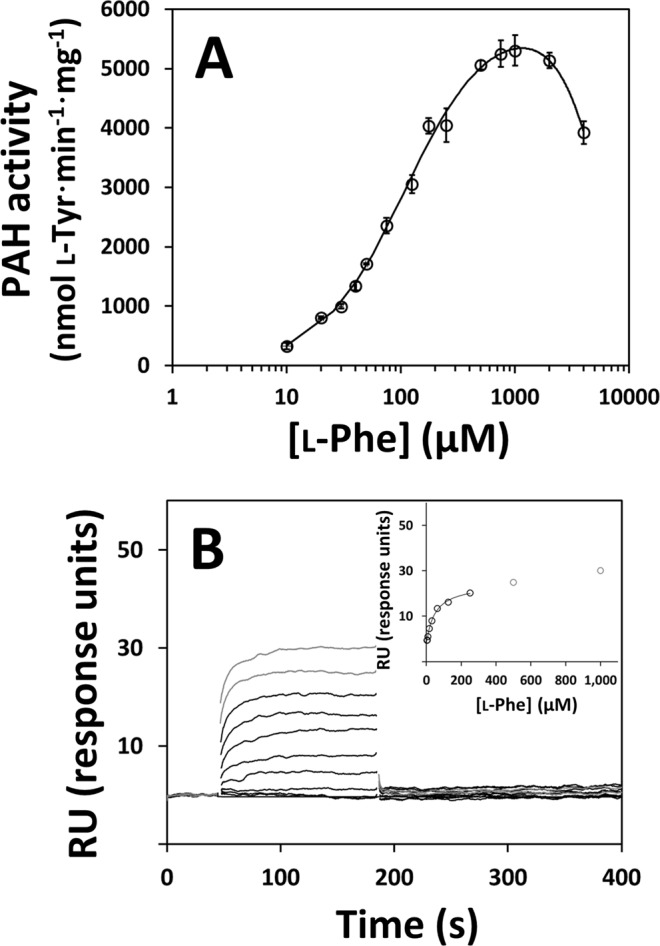
Kinetic characterisation of hPAH. *Panel A*, Effect of substrate concentration on the enzymatic activity of hPAH. Activity was measured at 25 °C in HEPES buffer pH 7.0 with 0.112 µM hPAH and 75 µM BH_4_. The obtained values for *V*_max_, *S*_0.5_, *h*, *K*_cat_/*S*_0.5_ and activation ratio are summarised in Supplementary Table 1. *Panel B*, Binding of l-Phe to hPAH determined by surface plasmon resonance. Inset, effect of l-Phe concentration on the steady-state response units.

The kinetic properties of N-terminally His_6_-tagged full-length tetrameric hPAH were analyzed by activity assays. hPAH activity was determined as a function of l-Phe concentration (Fig. 1A) and the data were fitted with a modified Hill equation accounting for substrate inhibition^31^, allowing to estimate the *V*_max_ (4688 ± 120 nmol l-Tyr·min^−1^·mg^−1^), *S*_0.5_ (107 ± 6 µM), *h* (1.8 ± 0.1) and catalytic efficiency (*K*_cat_/*S*_0.5_ = 3.01 µM^−1^·min^−1^). An activation ratio of 3.04 was calculated using the hPAH activities obtained from the pre-activated and non-activated assays (Supplementary Table 1).

The affinity of hPAH for l-Phe was also analyzed by surface plasmon resonance (SPR). As observed in the Inset to Fig. 1B, the steady-state RU appear to exhibit a slightly biphasic behavior, with the first phase being properly fitted up to 250 μM with an estimated steady-state affinity constant (*K*_D_) of 47 ± 8 μM. The experimental R_max_ estimated for the first phase of l-Phe binding per hPAH monomer was 1.7 ± 0.5 times higher than the theoretical R_max_. Taking into account the RU at the maximal l-Phe concentration (1 mM), the ratio between experimental RU and theoretical R_max_ is 1.9 ± 0.5, for a fully active immobilised surface. Moreover, the observed interaction is completely abolished in the presence of saturating concentrations of l-Phe (Supplementary Fig. S1), further validating the chip surface activity and excluding unspecific binding behavior of l-Phe to hPAH.

### Low-resolution structure of full-length human hPAH

**Figure 2:**
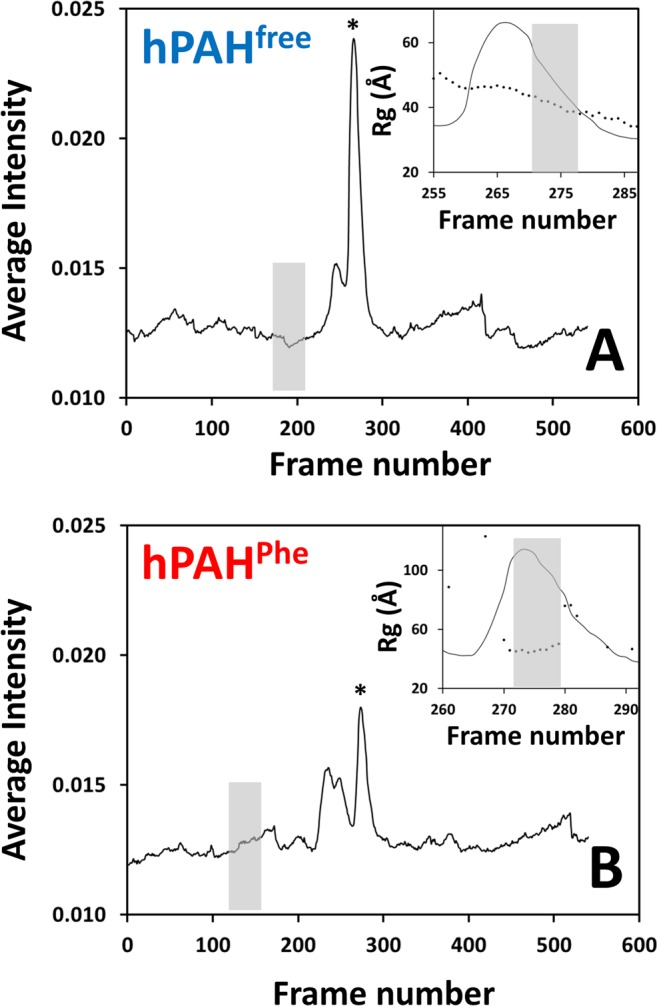
SEC-SAXS elution profiles of hPAH^free^ (non-incubated, panel A) and hPAH^Phe^ (incubated with 1 mM l-Phe, panel B). Frames are plotted in respect to the elution time. The size-exclusion chromatography column coupled to the beam allowed separating the tetramer peak (marked with *) from higher-order aggregates. Insets represent the *R*_*g*_ values for each frame along the tetramer peak. The sample frames were manually selected from the region where the *R*_*g*_ is constant. Buffer frames were selected using the “Buffer Automatic Selection” function of CHROMIXS.

We collected SAXS data on full-length hPAH in both the absence (hPAH^free^) and presence of 1 mM l-Phe (hPAH^Phe^) to characterise the conformational changes observed upon allosteric activation. Solutions of hPAH are typically inhomogeneous, with the protein adopting a number of oligomeric states and recombinant full-length hPAH forming large aggregates. Thus an online separation strategy was utilised with a size-exclusion chromatography column coupled to the SAXS set-up, facilitating isolation of the functional tetrameric form of hPAH for structural analysis. The observed elution profiles confirm the successful separation of oligomeric forms (Fig. 2).

**Figure 3:**
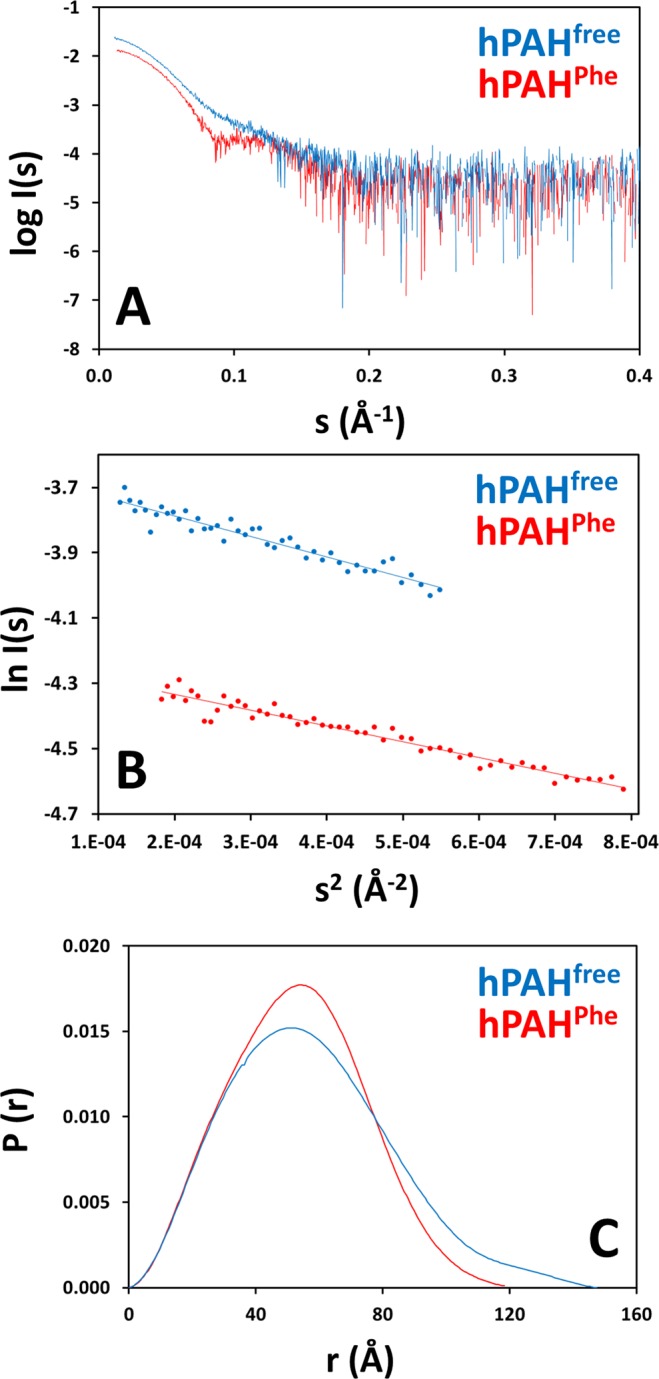
SAXS analysis of hPAH^free^ (non-incubated; blue) and hPAH^Phe^ (incubated with 1 mM l-Phe; red). *Panel A*, experimental scattering curves. A shift in the middle-*s* region suggests a structural difference between the free and Phe-bound protein consistent with domain rearrangement. *Panel B*, Guinier plots within the range of 0.50 < *sR*_*g*_ < 1.02 for hPAH^free^ and 0.52 < *sR*_*g*_ < 1.08 for hPAH^Phe^. Dots represent experimental points and lines represent linear regressions. No significant deviations from linearity are observed in the Guinier plot, thus no direct evidence of aggregation or polydispersity is observed. *Panel C*, pair-distribution functions *P(r)* derived from the scattering profiles. The *P(r)* function represents the sample in the real space. The differences in both curves suggest a conformational change upon addition of l-Phe: the loss of the extended tail at large r and the *D*_*max*_ decrease indicate a transition between an elongated conformation in hPAH^free^ and a compact conformation in hPAH^Phe^.

The SAXS profiles (Fig. 3A) and parameters extracted (Table 1) from the SEC-SAXS measurements of hPAH show that a significant change in structure occurs following exposure to l-Phe. The computed molecular mass determined from the experimental data supports the presence of a tetrameric species for the major peaks in both the free and bound forms (theoretical molecular mass ≈223 kDa), and the linearity of the Guinier plots (Fig. 3B) for *sR*_*g*_ < 1.1 provides confidence that the peak is not significantly contaminated by larger aggregates. Clear differences in the scattering profiles of the two samples are observed, providing direct evidence of distinct hPAH^free^ and hPAH^Phe^ conformations. The appearance of a minimum at *s* ~ 0.1 Å^−1^ for hPAH^Phe^ suggests that occupancy of the ligand binding site by l-Phe drives the tetramer toward a more rigid and potentially more compact structure. Indeed, the real-space distance distribution functions *P(r)* show compaction of the hPAH structure in the presence of l-Phe (Fig. 3C). hPAH^free^ displays a bell-shaped function with an extended tail at large distances *r*, indicative of a structure composed of a compact core and additional extended regions. hPAH^Phe^ displays a symmetric bell-shaped function without a significantly extended component, typical of a compact globular structure. In fact, the particle maximum distance, *D*_*max*_, is 24 Å shorter than that of the free protein. A reduction in the size of hPAH^Phe^ relative to hPAH^free^ is also supported by an observed ~4 Å decrease in the radius of gyration *R*_*g*_, again indicating that hPAH becomes more compact when ligand-bound.

**Table 1:** SAXS structural parameters of hPAH^free^ (non-incubated) and hPAH^Phe^ (1 mM l-Phe).

Sample	*R*_*g*_^Guinier^ (Å)	*R*_*g*_^*P*(r)^ (Å)	*D*_*max*_ (Å)	*V*_*P*_ (Å^3^)	MM (kDa)	MM_expected_ (kDa)
hPAH^free^	43.5 ± 0.37	44.4 ± 0.17	147	336,000	210	223
hPAH^Phe^	40.1 ± 0.37	40.4 ± 0.13	123	367,000	229	

Radii of gyration (*R*_*g*_) were estimated from the Guinier approximation and the pair-distribution function *P(r)*. Maximum particle dimensions (*D*_*max*_) were obtained from the pair-distribution function. Excluded particle volumes (*V*_*P*_) were estimated from the Porod approximation. Molecular mass (MM) values were derived from the Porod volume as MM = *V*_*P*_/1.6. The expected molecular mass (MM_expected_) was estimated based on the protein primary sequence. The experimental MMs are close to the expected and confirm the presence of tetrameric hPAH. The differences in *R*_*g*_ and *D*_*max*_ between the free- and bound-protein suggest a conformational change that brings the protein to a more compact conformation in the presence of ligand.

**Figure 4:**
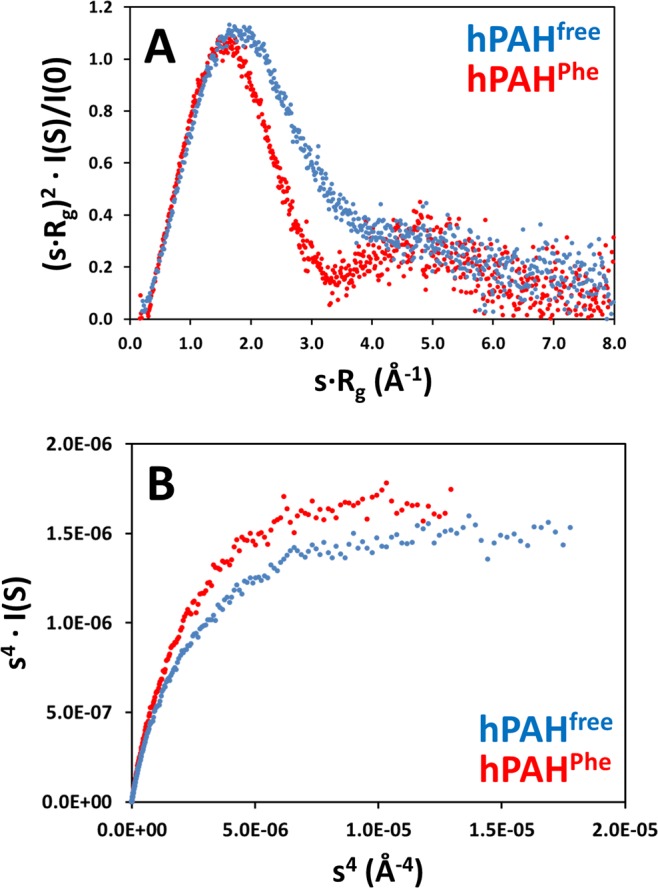
Analysis of flexibility versus conformational changes. *Panel A*, dimensionless Kratky plots (blue, non-incubated hPAH; red, l-Phe-incubated hPAH). *Panel B*, Porod-Debye plots limited by the *s* value corresponding to the major Kratky peak. Dots represent experimental data for hPAH^free^ (non-incubated; blue) and hPAH^Phe^ (1 mM l-Phe; red). The parabolic peak of the Kratky function with a maximum of 1.1 at *sR*_g_ = √3 and convergence to zero reveals folded proteins. The differences at high *s* between hPAH^free^ and hPAH^Phe^ suggest a conformational change that is confirmed by the Porod-Debye plot that shows two discrete plateaus.

The Kratky plots of hPAH^free^ and hPAH^Phe^ reveal folded proteins with non-significant flexibility, exhibiting parabolic peaks with maxima close to 1.1 at *sR*_g_ = √3 and that converge to zero^32^ (Fig. 4A). However, at higher angles the Kratky profiles diverge, with a more gradual decay to zero in the absence of l-Phe. Whether this is a result of two distinct conformations or of an increased relative flexibility for hPAH^free^ can be assessed by the Porod-Debye plot. The Porod-Debye approximation describes the decay of the scattering intensity as *s*^4^·*I(s)* vs. *s*^4^ ^33^. Folded particles, which have a clear scattering contrast, are predicted to display an asymptote at high *s* values. In flexible systems, the contrast between solvent and particle decreases and the asymptote is lost^33^. The clear and distinct plateaus of hPAH^free^ and hPAH^Phe^ (Fig. 4B) exclude destabilisation of the enzyme and confirm the existence of two distinct conformational states.

Low resolution structure determination was performed and the *ab initio* shapes reconstructed from the experimental data offer a hint about how domain rearrangement may occur in hPAH. A core of approximate dimensions of 92 × 88 × 56 Å for hPAH^free^ and 114 × 88 × 48 Å for hPAH^Phe^ easily accommodates a catalytic tetramer (determined as 100 × 78 × 42 Å from the hPAH_118–452_ crystal structure, PDB 2PAH^10^) (Supplementary Fig. S2). In both *ab initio* envelopes, peripheral volume can be occupied by the regulatory domains. These regions of scattering density differ in their relative position to the core: in hPAH^free^ two to four regions extend along the major axis that corresponds to the plane formed by the catalytic tetramer (the largest dimension increasing to ~136 Å); in hPAH^Phe^ two regions protrude along the minor axis, one above and one below the plane along the major axis (total height across the minor axis ~116 Å).

**Figure 5:**
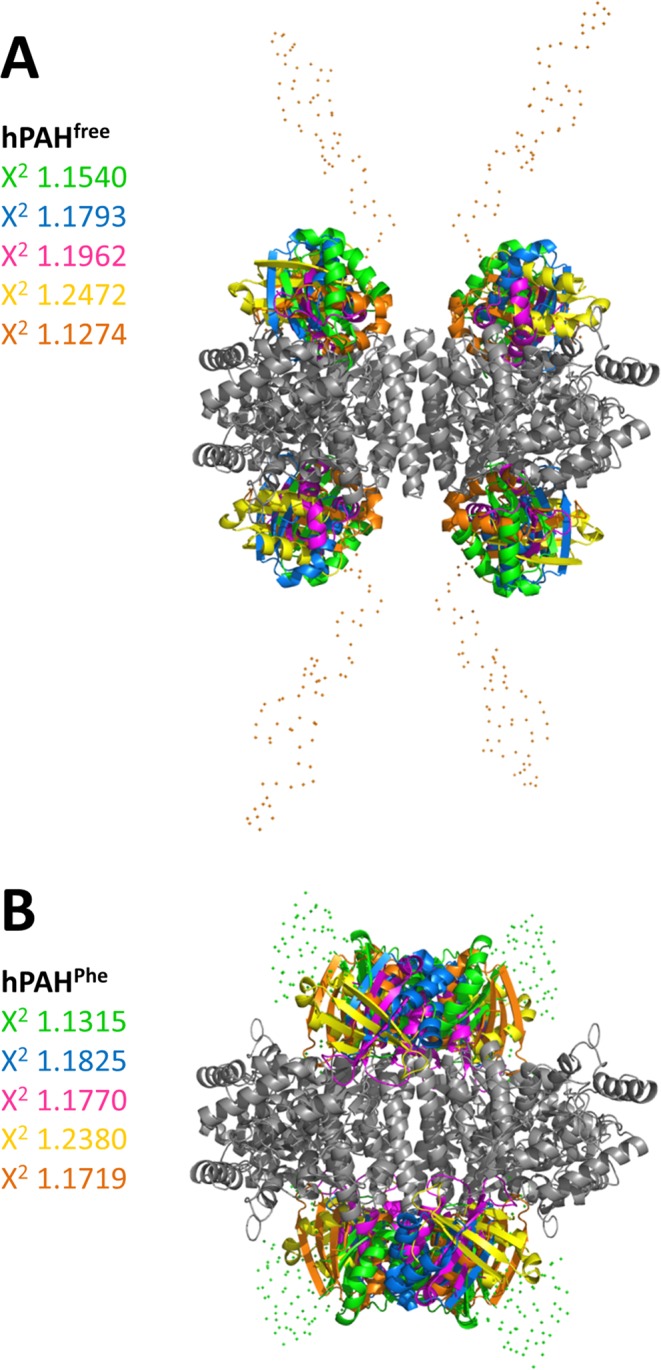
Evaluation of rigid-body modeling of hPAH^free^ (non-incubated; panel A) and hPAH^Phe^ (1 mM l-Phe; panel B). For each sample, five reconstructions were performed and superimposed to check for consistency of the models. In all models, tetramer cores (catalytic and oligomerisaton domains) are represented in gray. Regulatory domains are shown in a different color for each reconstruction. The chi-square of each model is displayed in its corresponding color. Dots represent the reconstruction of the N-terminus for the most representative model. The obtained structures from multiple rigid body refinements show identical position of domains, indicating reliability of the models.

**Figure 6:**
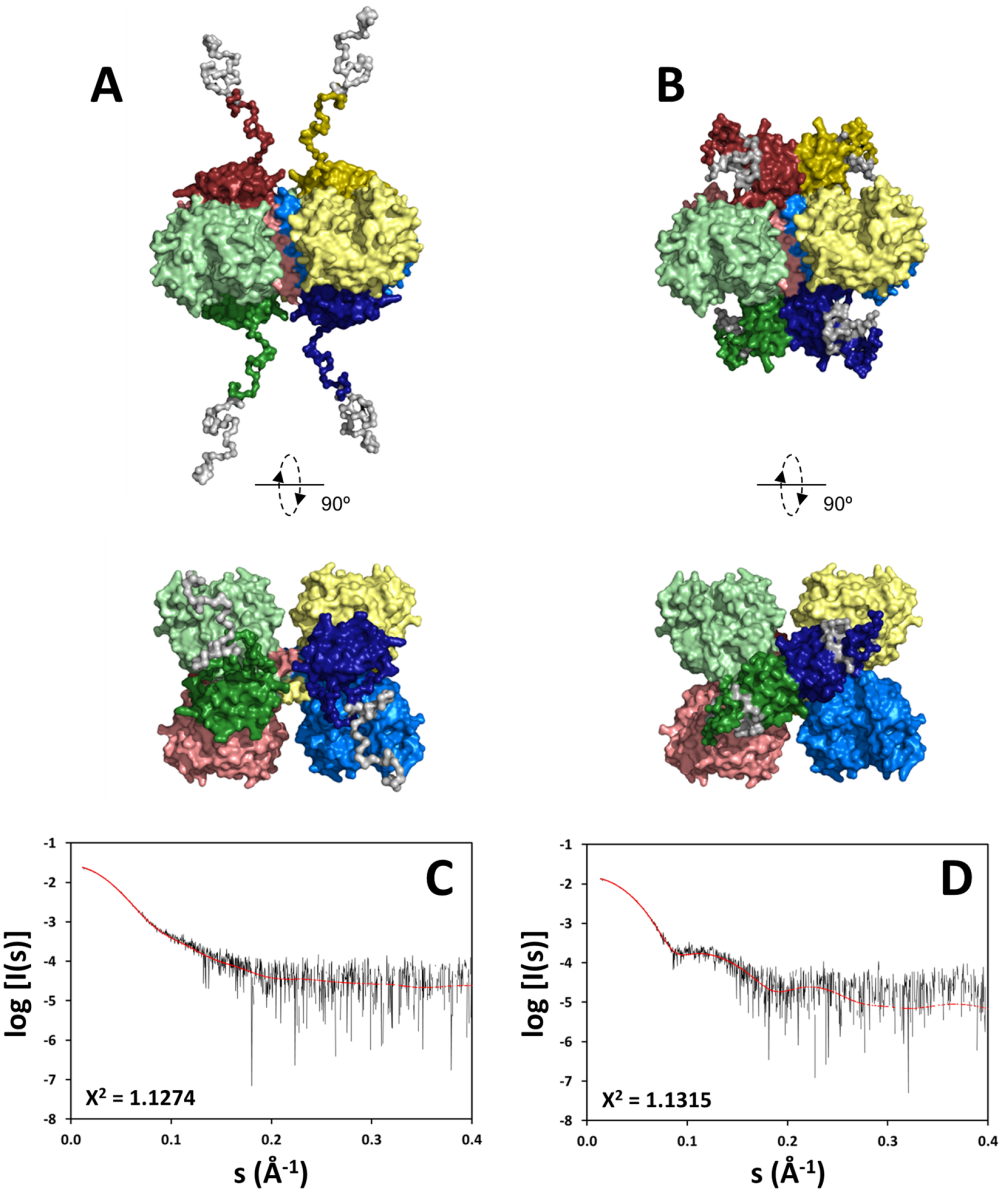
SAXS models of hPAH^free^ (non-incubated; panel A) and hPAH^Phe^ (1 mM l-Phe; panel B). Reconstruction was based on the coordinates of hPAH regulatory domain (PDB 5FII) and hPAH catalytic/oligomerisation domains (PDB 2PAH), using P222 symmetry. The models are shown in surface mode in two orientations, the bottom view being rotated 90° around the horizontal axis. Each color represents a monomer (regulatory domains are shown in darker tones). The scattering curves below each model (*panel C*, non-incubated hPAH; *panel D*, l-Phe-incubated hPAH) show the fit between experimental data (black line) and the corresponding model (red line). While the assembly of tetramer core is not altered by 1 mM l-Phe, the regulatory domains show a large-scale movement to form a dimeric structure above the four-helix bundle. This rearrangement explains the structural differences observed in the scattering profile of both hPAH states.

The overall shapes suggest a rearrangement of the regulatory domains upon allosteric activation by l-Phe. To determine the positions of regulatory domains in the absence and presence of l-Phe, we modeled the assembly of hPAH by rigid-body refinement. P222 symmetry restraints were applied to the tetramer. The high-resolution structures of the human regulatory, catalytic and oligomerisation domains were used for modeling, while missing regions (affinity tag, N-terminus and inter-domain linkers) were generated by CORAL and modeled as dummy residues. A number of independent reconstructions were performed for each hPAH form and in each case the structures calculated are highly consistent (Fig. 5), revealing two distinct conformational states. Figure 6 shows the two models that best fit the experimental data. The hPAH^free^ conformation in solution resembles that observed in the crystal structure of the rat protein (PDB 5DEN): separated regulatory domains are positioned above and below the plane of the catalytic tetramer. The N-terminal extensions (which include the affinity tag of our construct and the unstructured N-terminal tail of hPAH) protrude towards the solvent. The presence of l-Phe induces a repositioning of the regulatory domains, which adopt dimer-like structures above and below the four-helix bundle. The N-terminus is coiled around the regulatory domains.

### Effect of l-Phe on the global conformation and thermal stability of hPAH

**Figure 7:**
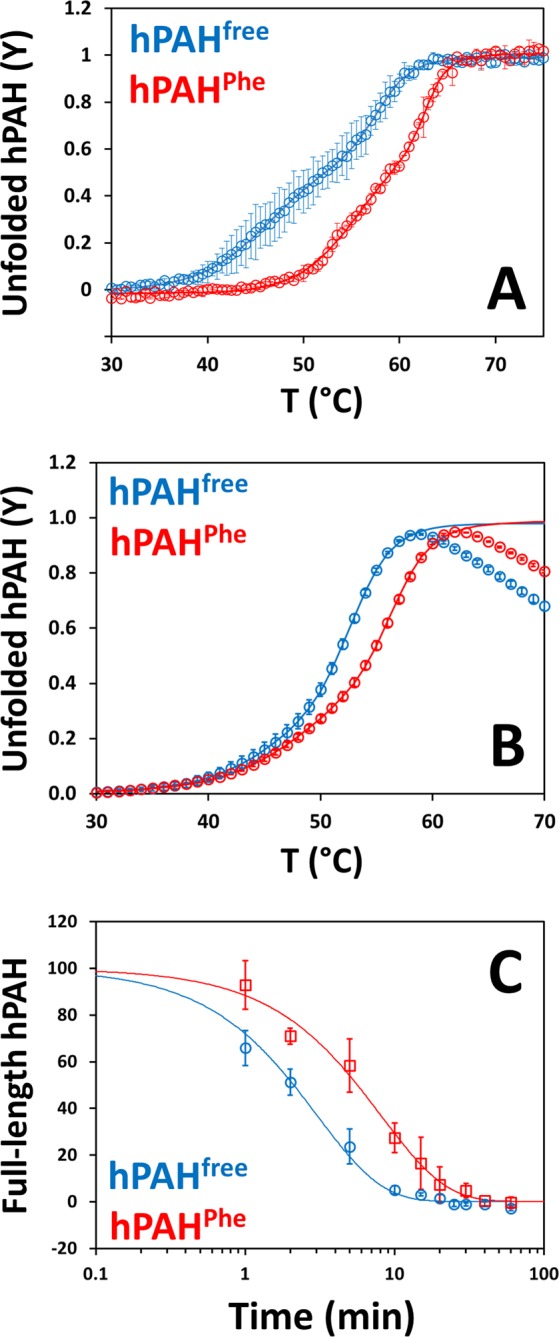
Response of hPAH^free^ (non-incubated; blue) and hPAH^Phe^ (1 mM l-Phe; red) to thermal denaturation and tryptic digestion. *Panel A*, thermal unfolding profile of hPAH as determined by far-UV CD. Each data point represents the mean of two independent assays and error bars represent the standard error. *Panel B*, thermal denaturation profiles of hPAH as determined by DSF. Both far-UV CD and DSF show two transitions that correspond to denaturation of the regulatory (first midpoint; *T*_m1_) and catalytic (second midpoint; *T*_m2_) domains. 1 mM l-Phe stabilises hPAH, resulting in an increase of both *T*_m_ values. *Panel C*, degradation of full-length hPAH by trypsin as a function of time. In each time point, the percentage of remaining full-length protein is normalised with respect to time 0 min. Each data point represents the mean of independent assays and error bars represent the standard error, where *n* = 4 for hPAH^free^ and *n* = 3 for hPAH^Phe^. The free enzyme is more susceptible to fast digestion. Conformational changes induced by l-Phe make hPAH more resistant to trypsin. The obtained melting temperatures (*T*_m_) and proteolytic rates (*k*_obs_) are summarised in Table 2.

Biophysical methodologies were herein employed to analyze the effect of incubation of full-length hPAH with 1 mM l-Phe on the protein’s conformational stability. Far-UV circular dichroism (CD) spectra of hPAH (*not shown*) exhibited a minimum at 222 nm, characteristic of proteins with high α-helical content. Upon thermal unfolding monitored by CD at 222 nm, hPAH displays a two-phase transition (Fig. 7A) best fitted with two *T*_m_ values that are significantly upshifted in the presence of 1 mM l-Phe (Table 2). Thermal denaturation assays monitored by differential scanning fluorimetry (DSF) yielded profiles which also displayed a two-phase transition (Fig. 7B) and were likewise best fitted with two *T*_m_ values that increase upon incubation with 1 mM l-Phe (Table 2). To analyze the global conformational changes of hPAH in the absence or presence of l-Phe, limited proteolysis by trypsin was analyzed as a function of time (Fig. 7C), allowing to estimate proteolytic rates (Table 2). Digestion of hPAH with trypsin leads to a progressive disappearance of the ≈56-kDa full-length band and concomitant formation of a ≈49-kDa band, as well as bands with lower molecular weights (Supplementary Fig. S3). As observed in Table 2, proteolytic rates indicate that the digestion of full-length hPAH is nearly 3 times slower in the presence of l-Phe as compared to the non-incubated protein. Intrinsic tryptophan fluorescence spectra were recorded for non-incubated and 1 mM l-Phe-incubated hPAH (Supplementary Fig. S4). The spectrum of non-incubated hPAH exhibits a broad band with λ_max_ ≈ 330 nm, where as incubation with 1 mM l-Phe leads to a slight decrease in the band intensity accompanied by a red-shift in λ_max_ to ≈340 nm.

**Table 2:** Effect of l-Phe on the thermal and proteolytic stability of hPAH.

Methodology	hPAH^free^	hPAH^Phe^
Far-UV CD	*T*_m1_: 46.0 ± 1.9 °C *T*_m2_: 57.5 ± 0.8 °C	*T*_m1_: 55.0 ± 0.4 °C *T*_m2_: 62.6 ± 0.3 °C
DSF	*T*_m1_: 46.6 ± 0.2 °C *T*_m2_: 52.8 ± 0.1 °C	*T*_m1_: 50.4 ± 0.2 °C *T*_m2_: 56.5 ± 0.1 °C
Limited proteolysis	*k*_obs_ = 0.33 ± 0.03 min^−1^	*k*_obs_ = 0.12 ± 0.01 min^−1^

Melting temperatures (*T*_m_) were determined by far-UV CD and DSF. Proteolytic rates (*k*_obs_) were obtained from trypsin limited proteolysis. Thermal stability is increased in the presence of 1 mM l-Phe. Resistance to proteolytic digestion is also increased by l-Phe.

## Discussion

The availability of structural data for full-length human phenylalanine hydroxylase has thus far been hampered by the inability to produce a conformationally homogeneous tetrameric preparation. Indeed, Flydal *et al*. recently reported a crystallographic structure of the three domains of hPAH, which required a truncation of the N-terminal thirteen amino acids to obtain a homogeneous conformation within the crystals^12^. Herein we employed an N-terminal His_6_-tag with a short linker to obtain a preparation amenable for the structural characterisation of full-length hPAH. In order to validate the structural data obtained in this work, a comparative analysis of the functional and biophysical properties of the fusion protein and the untagged hPAH (literature data) was carried out. The obtained data show an identical behavior indicating that the His_6_-tag does not influence hPAH function and structure. hPAH assembles as a tetramer that displays the expected kinetic parameters in terms of catalytic efficiency and positive cooperativity, retaining its regulatory properties namely l-Phe activation^16^. The thermal denaturation profile displays the characteristic two-phase transitions previously determined by differential scanning calorimetry and Far-UV CD^23^, characterised by a lower *T*_m1_ of 46 °C and a higher *T*_m2_ of 54 °C. These transitions have been attributed to the denaturation of regulatory and catalytic domains, respectively^23^. In accordance with these studies, hPAH is stabilised in the presence of 1 mM l-Phe, resulting in a shift of both transitions to higher temperatures.

The model of hPAH allosteric activation by l-Phe has been controversial on whether a local rearrangement around the catalytic site or a large-scale conformational change takes place. Studies supporting the latter model suggest that, similarly to rat PAH, activation of the hPAH involves repositioning and dimerisation of regulatory domains^11,13,15,19,29,30,34^. However, the lack of a structure of full-length activated human PAH has hampered validation of this model. Using small-angle X-ray scattering, we determined the low-resolution structures of the inactive (hPAH^free^) and active (hPAH^Phe^) states of full-length hPAH. Our structures validate the model of allosteric activation: that a large-scale movement and dimerisation of regulatory domains follows the cooperative binding of l-Phe.

The differences in scattering profiles and the distinct Porod-Debye plateaus for hPAH^free^ and hPAH^Phe^ confirm the existence of two discrete conformational states. Similar variations in the 0.05–0.2 Å^−1^ region have previously been observed for rat PAH^13,15^. For the rat enzyme, where crystal structures of the inactive state are available, these variations were attributed to a conformational transition: SAXS and crystallographic data agreed upon the free-protein conformation, while a mismatch was observed when the crystal structures were compared with the SAXS profile of the l-Phe-activated protein^13,15^. hPAH experiences a significant decrease in both *R*_*g*_ and *D*_*max*_ upon l-Phe binding, suggesting compaction of the protein. This is consistent with an l-Phe-specific change in the relative positions of hPAH domains. This compaction contrasts with the minor increase in *R*_*g*_ (40 Å to 41 Å) and the unchanged *D*_*max*_ (117 Å) observed for activated rat PAH^13,15^. The dimensions of rat PAH (both free and l-Phe-bound) are close to those of hPAH^Phe^, while hPAH^free^ dimensions suggest a more extended particle. These findings may result from differences inherent to the human and rat proteins, mainly due to the sequence divergence of their regulatory domain (that probably reflects in the observed differences in the response to l-Phe activation). Indeed, a comparative analysis of rat and human PAH by molecular dynamics simulations revealed a higher degree of flexibility for the human free protein, particularly in regulatory domains and C-terminal helices^12^. This flexibility may account for the occurrence of less compact conformations for hPAH^free^. Alternatively, the differences between SAXS parameters of rat and human PAH may arise from the N-terminus arrangement following repositioning of regulatory domains.

The overall assembly of the tetrameric core in our models is similar to the crystal structures of rat and human PAH (Supplementary Fig. S5): the C-terminal helices assemble in a four-helix bundle, forming a tetramer of catalytic domains. In the hPAH crystal structure, the tetramer is formed by a dimer of dimers through the two-fold crystallographic symmetry. The crystal structure of hPAH revealed that, within the dimer, a backbone variation in the region connecting the catalytic and oligomerisation domains results in a deviation from P222 symmetry^10^. This distortion was also observed in rat PAH: crystal structures (PDB codes 5DEN^13^, 5EGQ, 5FGJ^15^) revealed multiple conformations of the C-terminal helices, while a SAXS study suggested an even more pronounced asymmetry in solution^15^. Our initial attempts of modeling hPAH with rigid P222 symmetry yielded the expected tetrameric assembly, with minimal discrepancy to the experimental data, but with steric clashes at the C-terminal helices. By orienting the helices into a distorted configuration similar to the crystallographic observations, we were able to generate a model without structural artifacts. The flexibility of the C-terminal has been suggested to determine the existence of several conformational states, where variations in the helices’ positions allow for repositioning of domains during allosteric activation and catalysis^10,15,35^.

Apart from the presence of a 14-residue inter-domain linker, we did not impose upon CORAL any conformational restrictions for positioning the regulatory domains. Hence, their orientation in hPAH^free^ and hPAH^Phe^ differs between multiple reconstructions and from that observed for the rat L-Phe-free protein or the human dimerised regulatory domain (PDB 5FFI^11^) structures. Despite the uncertainty of the regulatory domains’ orientation, their relative position in the tetramer is consistent and unveils a transition from the inactive conformation (seen in rat PAH crystal structures) to a dimeric configuration of regulatory domains. It has been suggested that the N-terminal auto-inhibitory peptide may move away from the active site entrance upon rearrangement of the regulatory domains. However, this cannot be assessed from the current data and low-resolution models.

The transition from hPAH^free^ to hPAH^Phe^ explains some of the aforementioned biophysical observations. The differences between the SPR theoretical and experimental R_max_ had been previously observed by Stokka and Flatmark^18,22^. The authors proposed that a conformational transition, more than a ligand-binding event, would contribute to the obtained response for hPAH/l-Phe interactions. Knappskog and colleagues attributed the l-Phe-induced 10 nm red-shift on the emission maximum of hPAH to a change in Trp_120_ environment^20^. Structure analysis reveal that Trp_120_, located in the inter-domain linker, becomes more solvent exposed upon repositioning and dimerisation of the regulatory domains. This conformational transition also results in differential susceptibility to proteolysis. Two stages of cleavage can be observed: a fast initial cleavage, which results in a large fragment, and a subsequent slow cleavage, which yields a smaller fragment. Immunoblotting with anti-His antibody revealed that initial cleavage removes the N-terminal portion of full-length hPAH (*not shown*). In the presence of l-Phe, hPAH is more resistant to this stage, suggesting that association of regulatory domains protects against fast tryptic digestion probably by decreasing the accessibility of the hinge connecting the regulatory and catalytic domains (Arg_111_-Thr_117_: RDKKKDT) to trypsin digestion.

The low-resolution structure of a full-length hTH isoform (hTH1), one of the members of the AAAH family, was recently determined by SAXS^36^. hTH1 displays a similar assembly as hPAH^Phe^: catalytic and oligomerisation domains assembled in P222 symmetry form the tetrameric core, while regulatory domains form a dimer above the four-helix bundle. Just as for PAH, the dimeric configuration of regulatory domains in hTH1 produces a similar secondary maximum in the mid-*s* region of the scattering curve. Despite the near-identical overall structure, slight differences in dimensions reveal a more compact structure for hPAH than hTH1 (*R*_*g*_ = 47.4 Å; *D*_*max*_ = 200 Å). This is most likely explained as a difference in the flexibility of the C-terminal helices or the N-terminal peptide (twice as long in hTH1). Structural variations may also reflect different regulatory mechanisms of hPAH and hTH1, which is not pre-activated by its substrate but is physiologically inhibited by catecholamine derivatives. In opposition to hPAH, hTH1 regulatory domains dimerise both in the absence or presence of substrate^37^ and the scattering profile is identical in the absence and presence of inhibitor^36^. Although a conformational change is expected to block/release the active site entrance, local changes of the N-terminal peptide are predicted rather than a global motion of the regulatory domains as observed for hPAH.

## Methods

### hPAH expression and purification

Recombinant full-length human PAH was expressed as a hexa-histidine (His_6_) fusion protein with an N-terminal His_6_ tag and a 26-amino acid linker in *Escherichia coli* Top10 cells transformed with the pTrcHis-hPAH plasmid as described in^38^. Bacteria were grown in Luria-Bertani broth supplemented with 50 µg·mL^−1^ ampicillin at 37 °C. When OD_600_ reached 0.5, expression was induced by the addition of 1 mM isopropylthio-β-D-galactoside. Simultaneously, 0.2 mM ferrous ammonium sulfate (Fe^2+^) was added to the culture. After 3 h, at 37 °C, cells were harvested, resuspended in lysis buffer (50 mM sodium phosphate pH 7.8, 300 mM NaCl, 10% glycerol) supplemented with 1 mM phenylmethylsulfonyl fluoride, DNaseI and 1 mg·mL^−1^ lysozyme, and disrupted by three cycles of sonication during 60 sec at 50% duty free cycle (Media Cybernetics). After centrifugation at 13,000 g, 40 min, 4 °C, the soluble fraction was recovered for purification. An initial purification step was performed by immobilised metal affinity chromatography using a Ni-NTA resin (Qiagen) at 4 °C. The cell lysates were added to the resin pre-equilibrated in lysis buffer supplemented with 10 mM imidazole and stirred for 1 h at 4 °C. The resin was applied onto the column and washed in lysis buffer with a 20 to 75 mM imidazole gradient and hPAH was eluted with 250 mM imidazole. hPAH was further purified by size-exclusion chromatography using a HiLoad Superdex 200 HR column (GE Healthcare) in 20 mM Hepes pH 7.0, 200 mM NaCl (SEC buffer), at 4 °C. Protein batches were stored in liquid nitrogen. The isolated pure tetrameric form was employed in all biochemical and biophysical methods. Alternatively, for the SEC-SAXS measurements, the protein eluted from the Ni-NTA column and containing pure hPAH in its multiple oligomeric states was washed in a PD-10 column with SEC buffer to remove imidazole and applied directly onto the beamline SEC column.

### Enzymatic activity assays

Enzymatic activity was measured in 100 mM Hepes pH 7.0 in a final reaction volume of 200 µL essentially as described in^39^. The reaction mix was prepared with 5 µg His_6_-hPAH (corresponding to 0.112 µM of tetramer), 1 mM l-Phe (pre-activated condition) and 0.1 mg·mL^−1^ catalase and incubated for 4 min at 25 °C. Ferrous (Fe^2+^) ammonium sulfate (100 µM) was added and incubated for 1 min at 25 °C. The reaction was initiated by addition of 75 µM tetrahydrobiopterin (BH_4_). For determination of non-activated hPAH activity, the substrate l-Phe (1 mM) was added simultaneously with BH_4_. After 1 min, the reaction was stopped by adding 200 µL of cold 2% (v/v) acetic acid/ethanol solution, and the amount of produced l-Tyr was quantified by HPLC with fluorescence detection as in^39^. Specific activity is expressed in nmol of l-Tyr produced during 1 min per mg of protein (nmol l-Tyr·min^−1^·mg^−1^). The kinetic parameters were determined for the non-activated condition using variable concentrations of l-Phe (0–4 mM).

### Surface plasmon resonance

Binding of l-Phe to hPAH was evaluated by surface plasmon resonance (SPR) using a Biacore 4000 (GE Healthcare) instrument. The surface of a CM5 sensor chip was activated with 400 mM 1-ethyl-3-(3-dimethylaminopropyl)-carbodiimide and 100 mM *l*-hydroxysuccinimide for 10 min. hPAH (10 µg·mL^−1^ in 10 mM sodium acetate pH 5.5) was immobilised onto the activated chip using the standard amine coupling procedure. hPAH was coupled to the surface with a 1 to 2 min injection time at a flow rate of 10 µL·min^−1^ in order to reach 2,000 to 5,000 response units (RU). The free surface was blocked with a 7 min injection of 1 M ethanolamine (pH 8.5). l-Phe was directly dissolved in running buffer (10 mM Hepes pH 7.2, 150 mM NaCl, 5 mM MgCl_2_, 0.1 mM EDTA, 0.05% (v/v) Tween-20, 1 mM DTT) and injected at 10 different concentrations using a 2-fold dilution series, with the highest concentration tested being 1000 µM. Interaction analysis cycles consisted of a 140 s sample injection (30 µL·min^−1^; association phase) followed by 240 s of buffer flow (dissociation phase). All sensorgrams were processed by first subtracting the binding response recorded from the control surface (reference spot), followed by subtracting of the buffer blank injection from the reaction spot. All assays were performed at 25 °C. The interaction was assessed from the steady-state binding levels against l-Phe concentration, using the provided Biacore 4000 evaluation software (GE Healthcare).

### Small-angle X-ray scattering

Size-exclusion chromatography-coupled SAXS (SEC-SAXS) data were collected at B21 beamline at Diamond Light Source (Supplementary Table 2). hPAH samples (at 12 mg·mL^−1^; ≈214 µM in monomer) were injected in a Shodex KW-404 column using SEC buffer (equilibration and elution) at a flow-rate of 0.16 mL·min^−1^. For hPAH^Phe^ SAXS measurements, hPAH was pre-incubated with 1 mM l-Phe, which was also present in the SEC elution buffer. The estimated average tetramer concentration in the elution profile (Fig. 2B) is ≈15 µM, which implies ≈96% hPAH enzyme occupancy at this l-Phe concentration. Measurements were performed at 20 °C. Data were recorded using a Pilatus 2 M detector covering a range of momentum transfer 0.004 Å^−1^ ≤ s ≥ 0.408 Å^−1^ (s = 4πsinθ/λ, where 2θ is the scattering angle, and λ = 1.0 Å is the X-ray wavelength). Data were analyzed using the ATSAS program suite^40^. SEC-SAXS data were plotted with CHROMIXS^41^ and the sample frames were selected from the peak region where the radius of gyration (*R*_*g*_) is not influenced by aggregates. The final curves were generated after buffer subtraction (grey boxes in Fig. 2A,B). The radius of gyration (*R*_*g*_) was estimated from the Guinier approximation using PRIMUS^42^, and the maximum particle dimension (*D*_*max*_) from the pair distribution function *P(r)* using the GNOM package^43^. The molecular mass was estimated from the Porod volume. *Ab initio* shape determination was performed with DAMMIN^44^, DAMCLUST^45^ and the DAMAVER suite^46^: 20–30 dummy atom models were generated, clustered, aligned, averaged and refined to obtain the final model. Rigid body modeling was performed with CORAL^45^ using the structure coordinates of human catalytic/oligomerisation domain (PDB 2PAH) and human regulatory domain (PDB 5FII) as starting models. P222 symmetry was imposed for structure determination. The final structures were fit to the experimental data using CRYSOL^47^.

### Differential scanning fluorimetry

Thermal denaturation curves of hPAH were obtained by differential scanning fluorimetry (DSF). The reaction mix was prepared with 100 µg·mL^−1^ hPAH (corresponding to 0.45 µM of tetramer) and 2.5× Sypro Orange (Invitrogen; 5,000× commercially available stock solution). Thermal stability was measured between 20–90 °C with temperature increments of 1 °C·min^−1^. Fluorescence data were acquired in the FRET channel using a C1000 Touch thermal cycler equipped with a CFX96 optical reaction module (Bio Rad). Data were processed using CFX Manager Software V3.0 (Bio-Rad) and GraphPad Prism 6. The curves were fitted to a biphasic dose-response function and the melting temperatures of the regulatory (*T*_m1_) and catalytic (*T*_m2_) domains were obtained from the midpoint of the two transitions.

### Far-UV circular dichroism spectropolarimetry

Far-UV circular dichroism (Far-UV CD) spectra and thermal denaturation profiles were recorded in a Jasco J-710 spectropolarimeter, coupled to a Jasco PTC-348WI Peltier temperature controller and a Haake G/D8 water bath. Spectra of hPAH (at 250 μg·mL^−1^) in SEC buffer were measured in a 0.2 cm light path cuvette, and resulted from two accumulations at a scan rate of 50 nm·min^−1^, and a nitrogen flow of 6 L·min^−1^. Thermal denaturation profiles were obtained in the 10–90 °C temperature range, with a 1 °C·min^−1^ slope, and monitored at 222 nm (data pitch: 1 °C; delay time: 0 s; N_2_ flow: 3.5 L·min^−1^). Experimental data from thermal denaturation curves were fitted with a biphasic dose-response function and the *T*_m_ values were obtained from the midpoint of the first and second transitions.

### Intrinsic tryptophan fluorescence

Intrinsic tryptophan fluorescence (ITF) emission spectra were recorded in a Hitachi F-2000 spectrofluorimeter at 25 °C using λ_exc_ = 295 nm (slit 10 nm), λ_em_ between 305 and 500 nm (slit 10 nm) and a scan speed of 240 nm·min^−1^. Samples contained 20 μg·mL^−1^ of protein (final concentration) to maintain *A*_295_ < 0.02. The l-Phe effect was monitored by incubating the protein samples with 1 mM l-Phe for 5 min, at 25 °C, prior to ITF analysis.

### Limited proteolysis by trypsin

Limited proteolysis was performed at 25 °C in SEC buffer, using a trypsin:hPAH mass ratio of 1:200. Before cleavage, hPAH was incubated in the absence or presence of 1 mM l-Phe for 10 min at 25 °C. The reaction was initiated by addition of trypsin. At each time point, an aliquot was collected and the reaction stopped by addition of soybean trypsin inhibitor (at a trypsin:inhibitor mass ratio of 1:1.5) and 4× denaturing loading buffer. Samples were denatured for 5 min at 95 °C. The proteolytic profile was visualised by SDS-PAGE using 10% Bis-Tris precast gels (Invitrogen) ran with buffer 50 mM MOPS pH 7.7, 50 mM Tris, 0.1% SDS, 1 mM EDTA at 200 V. Gel bands were quantified with ImageJ and data were fitted to a single exponential decay equation using GraphPad Prism 6.

## Supplementary information

Supplementary Information

## Data Availability

The datasets generated during and/or analysed during the current study are available from the corresponding authors on reasonable request.
