# Author Correction: Structure of full-length wild-type human phenylalanine hydroxylase by small angle X-ray scattering reveals substrate-induced conformational stability

**DOI:** 10.1038/s41598-019-54425-2

**Published:** 2019-11-20

**Authors:** Catarina S. Tomé, Raquel R. Lopes, Pedro M. F. Sousa, Mariana P. Amaro, João Leandro, Haydyn D. T. Mertens, Paula Leandro, João B. Vicente

**Affiliations:** 1 Instituto de Tecnologia Química e Biológica António Xavier, Universidade Nova de Lisboa, Oeiras, Portugal; 2 Instituto de Biologia Experimental e Tecnológica, Oeiras, Portugal; 3 Research Institute for Medicines (iMed.ULisboa) and Department of Biochemistry and Human Biology, Faculty of Pharmacy, Universidade de Lisboa, Lisbon, Portugal; 4 EMBL Hamburg c/o DESY, Hamburg, Germany; 5 Present Address: Department of Genetics and Genomic Sciences and Icahn Institute for Data Science and Genomic Technology, Icahn School of Medicine at Mount Sinai, New York, NY USA

Correction to: *Scientific Reports* 10.1038/s41598-019-49944-x, published online 20 September 2019

The authors would like to acknowledge the missing reference of Arturo and colleagues publication in the original version of the Article, which describes structural data for a full-length p.C29S PAH variant in its l-Phe-activated form. This paper is cited here as Ref. 1 and should have been included in the text as below.

In the Introduction, the paragraph,

“Structural analyses of hPAH have relied on crystal structures of truncated forms of the enzyme lacking one or two domains^5–12^ and full-length bound to BH_4_^12^, and on crystallographic and SAXS structures of the full-length rat homologue^13–15^.”

should read:

“Structural analyses of hPAH have relied on crystal structures of truncated forms of the enzyme lacking one or two domains^5–12^ and full-length bound to BH_4_^12^, and on crystallographic and SAXS structures of the full-length C29S variant form of hPAH^1^ and full-length rat homologue^13–15^.”

In the same section, the paragraph,

“Solution structural analyses of rat PAH confirmed distinct conformations for the inactive and l-Phe-activated enzymes and supported dimerisation of regulatory domains as the substrate activation mechanism^13,15^.”

should read

“Solution structural analyses of rat PAH and hPAH C29S variant confirmed distinct conformations for the inactive and l-Phe-activated enzymes and supported dimerisation of regulatory domains as the substrate activation mechanism^1,13,15^.”

In the Discussion,

“Similar variations in the 0.05–0.2 Å–1 region have previously been observed for rat PAH^13,15^.”

should read:

“Similar variations in the 0.05–0.2 Å–1 region have previously been observed for rat PAH^13,15^ and, more recently, for the hPAH C29S variant^1^.”

This correction does not alter the main conclusions of the Article.

## References

[1] Arturo EC, Gupta K, Hansen MR, Borne E, Jaffe EK (2019). Biophysical characterization of full-length human phenylalanine hydroxylase provides a deeper understanding of its quaternary structure equilibrium. J Biol Chem.

