# Supplementary material for: Structure of full-length wild-type human phenylalanine hydroxylase by small angle X-ray scattering reveals substrate-induced conformational stability
Source: Sci Rep. 2019 Sep 20;9:13615. doi: 10.1038/s41598-019-49944-x (PMC6754429; doi:10.1038/s41598-019-49944-x)
Supplement: Supplementary file 1 — Supplementary Information [file 41598_2019_49944_MOESM1_ESM.pdf]

SUPPORTING INFORMATION

Structure of full-length wild-type human phenylalanine hydroxylase by small angle X-ray scattering reveals substrate-induced conformational stability

Catarina S. Tomé, Raquel R. Lopes, Pedro M. F. Sousa, Mariana P. Amaro, João Leandro, Haydyn D. T. Mertens, Paula Leandro and João B. Vicente

Page	Name	Description
S2	Supplementary Table 1	Kinetic parameters of hPAH.
S3	Supplementary Table 2	Small-angle X-ray scattering data collection and experimental parameters for $hPAH^{free}$ (non-incubated) and $hPAH^{Phe}$ (1 mM L-Phe).
S5	Supplementary Figure S1	SPR competition analysis of hPAH interaction with L-Phe.
S6	Supplementary Figure S2	*Ab initio* shape reconstructions of (A) $hPAH^{free}$ (non-incubated) and (B) $hPAH^{Phe}$ (1 mM L-Phe)
S7	Supplementary Figure S3	SDS-PAGE analysis of the tryptic digestion of $hPAH^{free}$ (non-incubated) and $hPAH^{Phe}$ (1 mM L-Phe)
	Supplementary Figure S4	Fluorescence emission spectra of $hPAH^{free}$ (non-incubated) and $hPAH^{Phe}$ (1 mM L-Phe)
S8	Supplementary Figure S5	Superimposition of the crystal and solution structures of tetrameric hPAH catalytic core
S9	Supplementary Figure S6	Original scan of gel from SDS-PAGE analysis of the tryptic digestion of hPAHfree (non-incubated) and hPAHPhe (1 mM L-Phe) in Supplementary Figure S3
S10	References	

Supplementary Table 1. Kinetic parameters of hPAH. The N-terminal His_6-tagged construct of human phenylalanine hydroxylase displays identical parameters to those reported in the literature, namely maximum velocity (V_{max}), L-Phe concentration at half-maximal activity ($S_{0.5}$), L-Phe cooperativity (h), catalytic efficiency ($K_{cat}/S_{0.5}$) and activation ratio. Values from the untagged enzyme [1] are presented as a reference.

Sample	V_{max} (nmol Tyr·min^{-1}·mg^{-1})	$S_{0.5}$ (μM)	h	$K_{cat}/S_{0.5}$ (μM^{-1}·min^{-1})	Activation ratio
His_6-tagged hPAH	4688 ± 120	107 ± 6	1.8 ± 0.1	3.01	3.04
Untagged hPAH	2790 ± 70	151 ± 6	2.1 ± 0.1	4.00	3.60

Supplementary Table 2. Small-angle X-ray scattering data collection and experimental parameters for hPAH^{free} (non-incubated) and hPAH^{Phe} (1 mM L-Phe).

(*a*) Sample details

	hPAH^{free}	hPAH^{Phe}
Organism	*Homo sapiens*	*Homo sapiens*
Source	*E. coli* Top10	*E. coli* Top10
UniProt sequence ID (residues in construct)	P00439 (1–452)*	P00439 (1–452)*
Extinction coefficient ε (280 nm, 0.1% w/v)	0.910	0.910
Partial specific volume $\bar{\upsilon}$ ($cm^3 \cdot g^{-1}$)	0.738	0.738
Mean solute and solvent scattering length densities and mean scattering contrast $\Delta\bar{\rho}$ ($\rho_{protein}$-$\rho_{solvent}$) (10^{10} cm^{-2})	2.82 (12.29-9.48)	2.80 (12.28-9.48)
Molecular mass *M* from chemical composition (tetramer) (Da)	223 000	223 000
SEC-SAXS, Shodex KW-404 column		
Concentration ($mg \cdot ml^{-1}$) [A_{280nm}]	12.0	12.0
Injection volume	45 µL	45 µL
Flow rate ($mL \cdot min^{-1}$)	0.16	0.16
Solvent composition	20 mM Hepes, 200 mM NaCl, pH 7.0	

(*b*) SAS data collection parameters

Instrument/Data processing	B21, Diamond Light Source (DLS), Harwell, (UK)
Wavelength (Å)	1.0
Beam geometry (size, sample-to-detector distance)	1 × 1 mm, 4 m
s-measurement range ($Å^{-1}$)	0.004-0.408
Absolute scaling method	Comparison with scattering from protein standard (BSA)
Basis for normalization to constant counts	To the integrated intensity from the beam-stop diode
Method for monitoring radiation damage	Frame comparison
Exposure time, number of exposures	1620 s (540 × 3.0 s)
Sample temperature (ºC)	20

(*c*) Software employed for SAS data reduction, analysis and interpretation

SAS data reduction	*DAWN* pipeline (DLS, Harwell, UK), *CHROMIXS* [2]
Calculation of ε from sequence	*ProtParam* [3]
Calculation of $\Delta\bar{\rho}$ and $\bar{\upsilon}$ values from chemical composition	*MULCh* 1.1 [4]
Basic analyses: Guinier, *P*(*r*), scattering particle volume (V_P)	*PRIMUSqt* from ATSAS 2.8.3 [5]
Shape/bead modelling	DAMMIN [6], DAMCLUST [7] and DAMAVER suite [8]
Atomic structure modelling	CORAL [7]
Molecular graphics	PyMOL v1.7.2.1

(*d*) Structural parameters #		
	hPAH^{free}	hPAH^{Phe}
Guinier Analysis		
$I(0)$ (Rel. Units)	0.026 ± 0.00015	0.015 ± 0.0001
R_g (Å)	43.48 ± 0.37	40.06 ± 0.37
s-range (Å^{-1})	0.011-0.030	0.013-0.032
$P(r)$ analysis		
$I(0)$ (Rel. Units)	0.026 ± 0.00010	0.015 ± 0.00006
R_g (Å)	44.4 ± 0.17	40.4 ± 0.13
d_{max} (Å)	147 ± 5	123 ± 5
s-range (Å^{-1})	0.011-0.184	0.014-0.200
χ^2 (total estimate from *GNOM*)	0.92	0.83
M_r from (V_P) (Da)	210,000	229,000
Volume (V_P) (Å^3)	336,000	367,000

(*e*) Shape modelling results		
	hPAH^{free}	hPAH^{Phe}
DAMMIN (default parameters, 20-30 calculations)		
s-range for fitting (Å^{-1})	0.011-0.184	0.014-0.200
Symmetry, anisotropy assumptions	P222, oblate	P222, none
NSD (standard deviation)	0.63 (cluster: 0.60)	0.78 (cluster: 0.63)
χ^2 value/range	1.015-1.023	1.155-1.160
Constant adjustment to intensities	1.726×10^{-5}	5.135×10^{-8}
P value	0.140621	0.039866
M_r estimate as 0.5 × volume of models (Da)	193,500	175,700
Model resolution from *SASRES* [9] (Å)	43 ± 3	49 ± 4

(*f*) Atomistic modelling		
	hPAH^{free}	hPAH^{Phe}
CORAL rigid body modeling		
Starting crystal structures	2PAH:A ; 5FII:A	2PAH:A ; 5FII:A
Flexible residues	1-34, 110-124	1-34, 110-124
s-range for fitting (Å)	0.0134-0.4077	0.0135-0.4077
Symmetry, anisometry assumptions	P222, none	P222, none
χ^2, *CORMAP P* value	1.1274, 0.021	1.1315, 0.083

(*g*) SASBDB IDs for data and models	
hPAH^{free}	hPAH^{Phe}
Draft ID 1578 (submitted)	Draft ID 1580 (submitted)

* The construct contains 36 additional residues from the N-terminal His_6 tag.
# The experimental curves were obtained using frames 271-277 (hPAH^{free}) and 272-279 (hPAH^{Phe}).

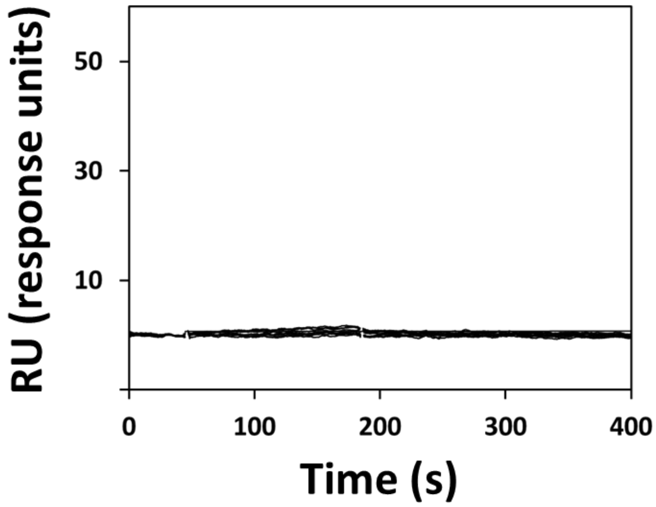

Supplementary Figure S1. SPR competition analysis of hPAH interaction with L-Phe. Binding of L-Phe to hPAH was analyzed in presence of 1000 μM L-Phe. L-Phe was injected at 10 different concentrations using a 2-fold dilution series (highest concentration 1000 μM), for 140 s followed by 240 s of buffer injection.

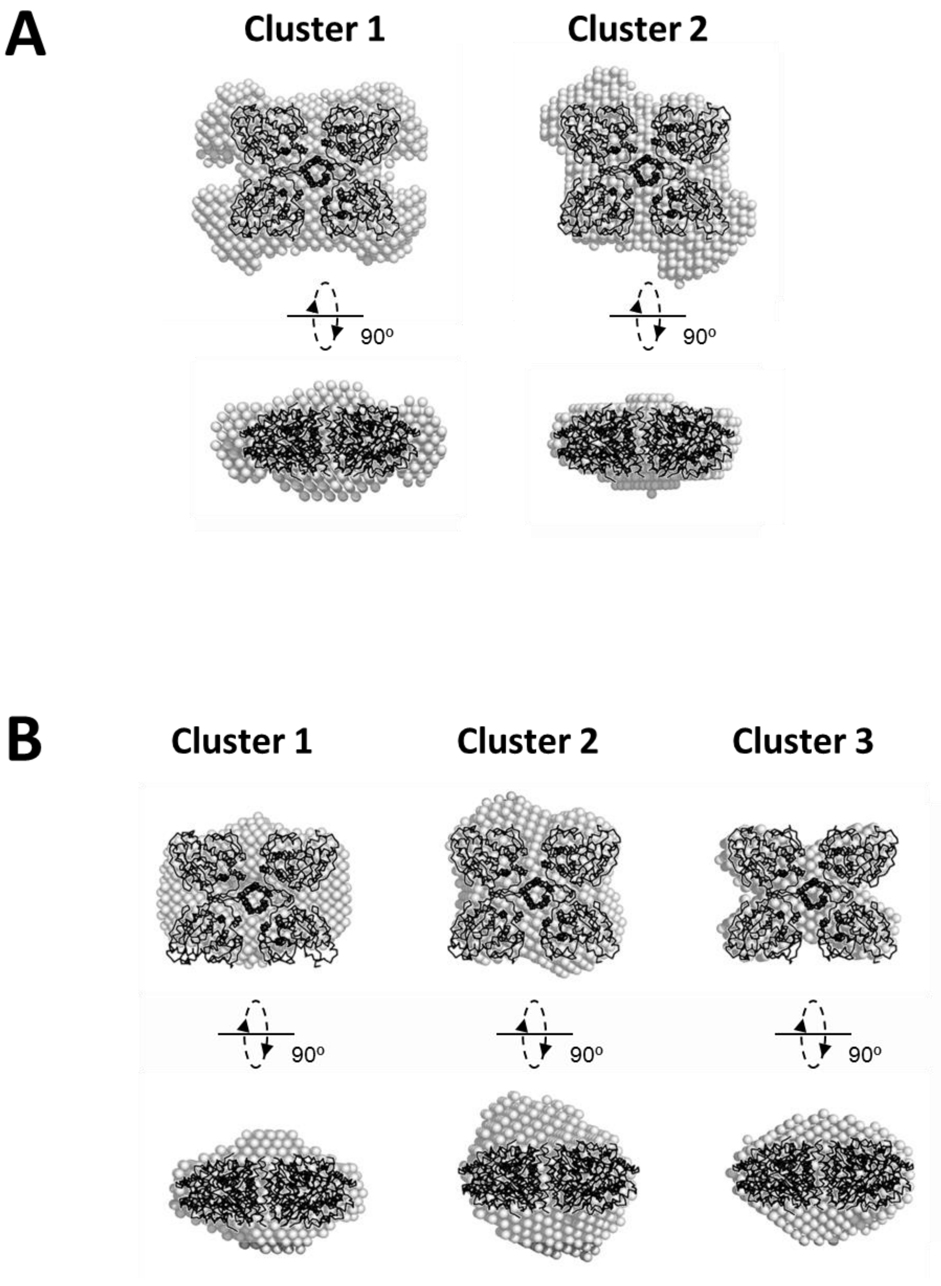

Supplementary Figure S2. ***Ab initio*** **shape reconstructions of (A) hPAH^{free} (non-incubated) and (B) hPAH^{Phe} (1 mM L-Phe).** For each sample, the generated dummy atom models were clustered with DAMCLUST [7] into groups containing similar models. Two clusters for hPAH^{free} and three clusters for hPAH^{Phe} were obtained. The displayed models (in gray spheres) correspond to the final refined *ab initio* shapes. The catalytic core of hPAH (PDB 2PAH) is represented in black ribbons and superimposed on the SAXS reconstructions. In all models, the tetrameric catalytic domains properly fit the density. In hPAH^{free} reconstructions, remaining density is present in the vicinity of the core plane. In hPAH^{Phe} reconstructions, extra density is present above and below the catalytic plane, suggesting a different positioning of regulatory domains, closer to the extremities of the four-helix bundle.

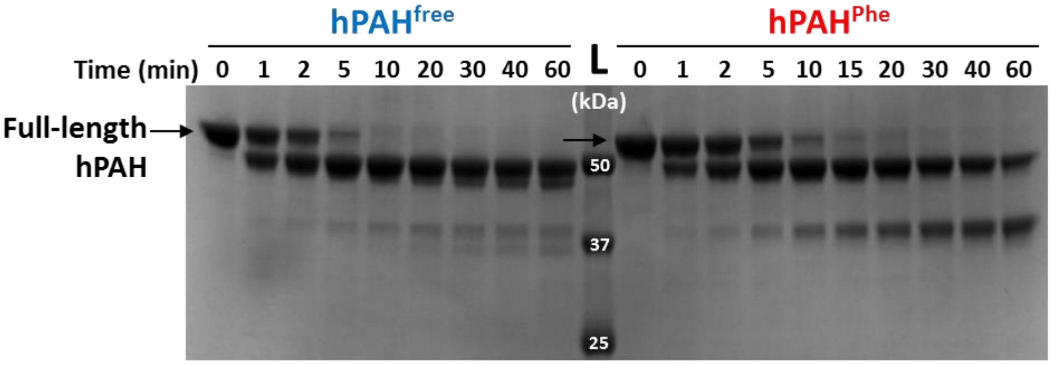

Supplementary Figure S3. SDS-PAGE analysis of the tryptic digestion of hPAH^{free} (non-incubated) and hPAH^{Phe} (1 mM L-Phe). Each lane contains an aliquot of the reaction, corresponding to a time point of the proteolytic digestion (from 0 min to 60 min, as indicated above the gel). The full-length protein is highlighted by an arrow. L indicates the molecular weight marker. Gel cropped to highlight bands of interest; uncropped gel in Supplementary Figure S6.

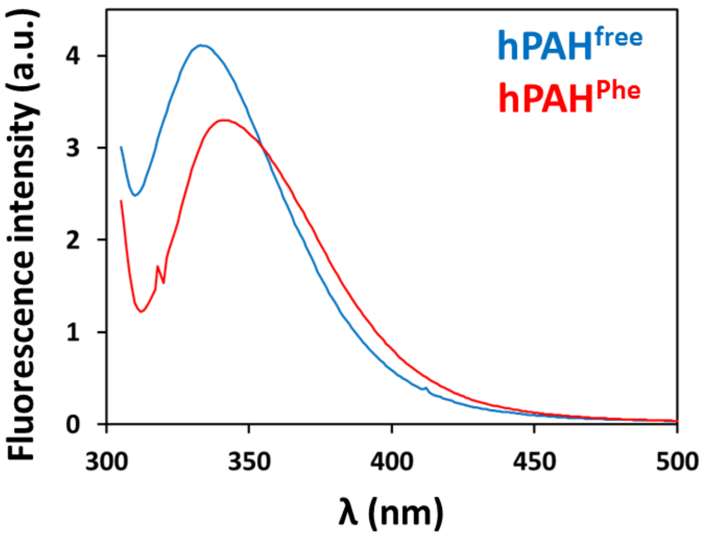

Supplementary Figure S4. Fluorescence emission spectra of hPAH^{free} (non-incubated) and hPAH^{Phe} (1 mM L-Phe). L-Phe produces a +10 nm shift in the emission maximum of hPAH, attributed to increased Trp120 fluorescence.

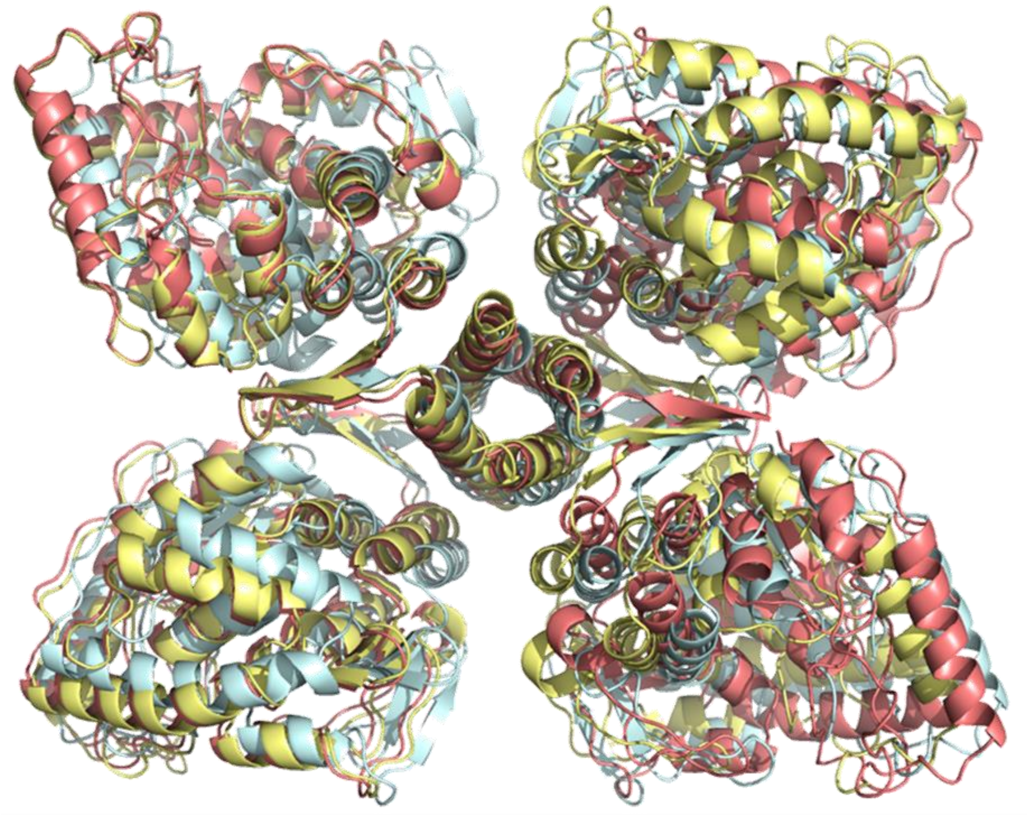

Supplementary Figure S5. Superimposition of the crystal and solution structures of tetrameric hPAH catalytic core. Human $PAH_{118\text{-}452}$ crystal structure (PDB 2PAH) is represented in yellow. Rat $PAH_{22\text{-}449}$ crystal structure (PDB 5DEN; here truncated to 118-449) is represented in blue. Solution structure of human PAH ($hPAH^{free}$, residues 124-452) determined by SAXS is represented in pink (the catalytic core of $hPAH^{Phe}$ is identical to that of $hPAH^{free}$ and thus was omitted from the figure). The rearrangement of human PAH catalytic/oligomerization domains in solution is similar to the crystal structures of human and rat PAH. Slight differences between SAXS and crystal structures account for variations in orientation of the C-terminal helices.

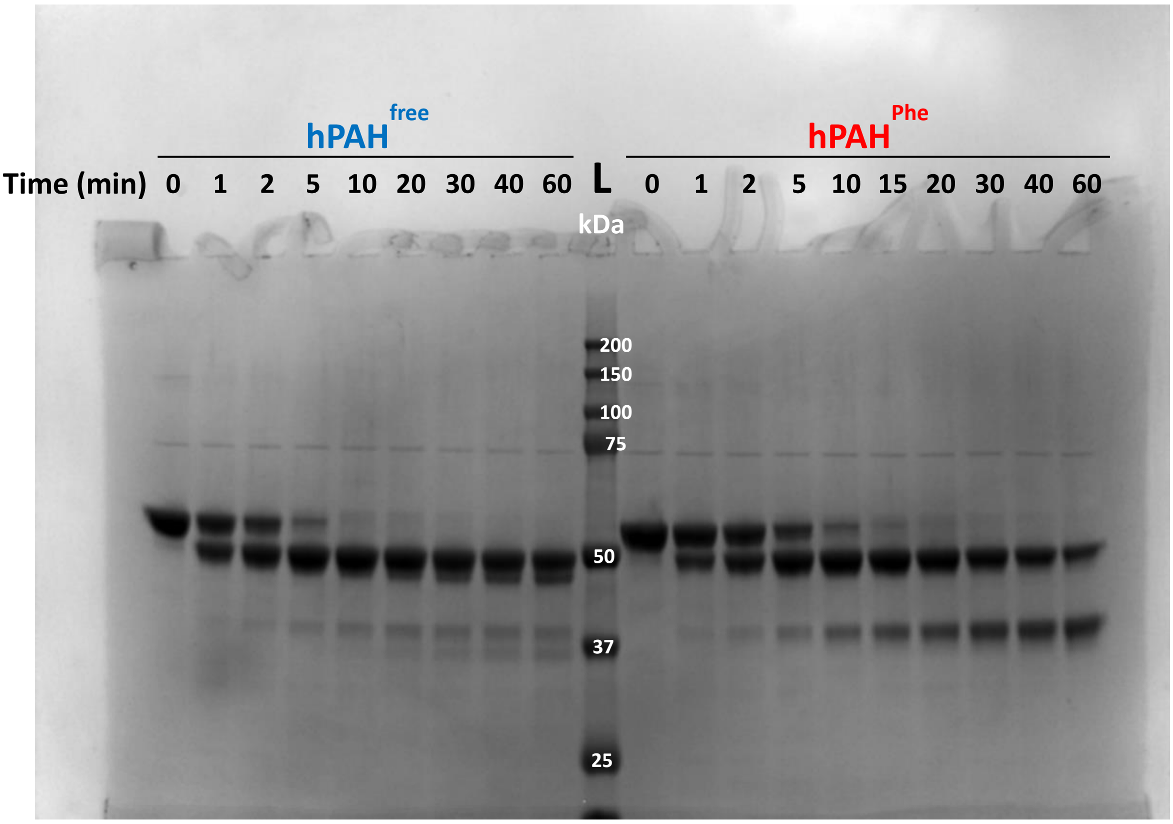

Supplementary Figure S6. Original scan of gel from SDS-PAGE analysis of the tryptic digestion of hPAH^{free} (non-incubated) and hPAH^{Phe} (1 mM L-Phe) in Supplementary Figure S3.

References

1. Leandro, J., Leandro, P., Flatmark, T. Heterotetrameric forms of human phenylalanine hydroxylase: co-expression of wild-type and mutant forms in a bicistronic system. *Biochim Biophys Acta.* **1812**(5), 602-12 (2011).
2. Panjkovich, A., Svergun, D. I. CHROMIXS: automatic and interactive analysis of chromatography-coupled small-angle X-ray scattering data. *Bioinformatics.* **34**(11), 1944-6 (2018).
3. Gasteiger, E., *et al.* Protein Identification and Analysis Tools on the ExPASy Server. In: Walker JM, editor. The Proteomics Protocols Handbook. Totowa, NJ: Humana Press; 2005. p. 571-607.
4. Whitten, S. T., Garcia-Moreno, B. E., Hilser, V. J. Ligand effects on the protein ensemble: unifying the descriptions of ligand binding, local conformational fluctuations, and protein stability. *Methods Cell Biol.* **84**, 871-91 (2008).
5. Konarev, P. V., Volkov, V. V., Sokolova, A. V., Koch, M. H. J., Svergun, D. I. PRIMUS: a Windows PC-based system for small-angle scattering data analysis. *Journal of Applied Crystallography.* **36**, 1277-82 (2003).
6. Svergun, D. I. Restoring low resolution structure of biological macromolecules from solution scattering using simulated annealing. *Biophys J.* **76**(6), 2879-86 (1999).
7. Petoukhov, M. V., *et al.* New developments in the ATSAS program package for small-angle scattering data analysis. *J Appl Crystallogr.* **45**(Pt 2), 342-50 (2012).
8. Volkov, V. V., Svergun, D. I. Uniqueness of ab initio shape determination in small-angle scattering. *Journal of Applied Crystallography.* **36**, 860-4 (2003).
9. Tuukkanen, A. T., Kleywegt, G. J., and Svergun, D. I. Resolution of ab initio shapes determined from small-angle scattering. *IUCrJ* **3**, 440-447 (2016).
